# Effects of system response delays on elderly humans’ cognitive performance in a virtual training scenario

**DOI:** 10.1038/s41598-019-44718-x

**Published:** 2019-06-05

**Authors:** Maria Wirzberger, René Schmidt, Maria Georgi, Wolfram Hardt, Guido Brunnett, Günter Daniel Rey

**Affiliations:** 1 Max Planck Research Group “Rationality Enhancement”, Max Planck Institute for Intelligent Systems, Max-Planck-Ring 4, 72076 Tübingen, Germany; 2 Computer Engineering, Faculty of Computer Science, TU Chemnitz, Straße der Nationen 62, 09111 Chemnitz, Germany; 3 Information Science, Faculty of Languages, Literature and Culture, University of Regensburg, Universitätsstraße 31, 93053 Regensburg, Germany; 4 Computer Graphics and Visualization, Faculty of Computer Science, TU Chemnitz, Straße der Nationen 62, 09111 Chemnitz, Germany; 5 Psychology of Learning with Digital Media, Faculty of Humanities, TU Chemnitz, Straße der Nationen 12, 09111 Chemnitz, Germany

**Keywords:** Human behaviour, Computer science

## Abstract

Observed influences of system response delay in spoken human-machine dialogues are rather ambiguous and mainly focus on perceived system quality. Studies that systematically inspect effects on cognitive performance are still lacking, and effects of individual characteristics are also often neglected. Building on benefits of cognitive training for decelerating cognitive decline, this Wizard-of-Oz study addresses both issues by testing 62 elderly participants in a dialogue-based memory training with a virtual agent. Participants acquired the method of loci with fading instructional guidance and applied it afterward to memorizing and recalling lists of German nouns. System response delays were randomly assigned, and training performance was included as potential mediator. Participants’ age, gender, and subscales of affinity for technology (enthusiasm, competence, positive and negative perception of technology) were inspected as potential moderators. The results indicated positive effects on recall performance with higher training performance, female gender, and less negative perception of technology. Additionally, memory retention and facets of affinity for technology moderated increasing system response delays. Participants also provided higher ratings in perceived system quality with higher enthusiasm for technology but reported increasing frustration with a more positive perception of technology. Potential explanations and implications for the design of spoken dialogue systems are discussed.

## Introduction

With an estimated 2 billion users and expected annual revenues of nearly $16 billion in 2021^1,2^, intelligent voice assistants, such as Google Home, Alexa, Siri, and Mycroft AI, are becoming increasingly relevant. The trend towards embedding such technologies in mobile systems, such as smartphones and tablets, will eventually make this technology omnipresent. However, while mobility is a huge benefit, limited hardware resources and increased computing time cause delays in system reactions that could decrease users’ acceptance of the resulting system.

So far, studies on the effects of system response delays are rather scarce^3^ and controversial. For instance^4^, reported that participants rated a system with 0.60 s faster reaction time as politer, more efficient and more transparent. By contrast, a study by^5^ showed that a dialogue system with a very short response time (*M* = 0.37 s) received worse ratings. Therefore, the system-induced delay might not have been the only relevant factor in these contexts, and future research should examine a framework of related factors. In computer-based learning and training scenarios, it is further necessary to examine whether the length of the system response delay also affects cognitive performance. Although this issue has received little attention, research on task interruptions (e.g.^6^) makes it possible to hypothesise that performance will decrease as the system response delay increases.

With a first exploratory study that used a simulated computer-based memory training with an animated virtual agent^7^, demonstrated that longer system response delays decreased recall performance. These effects were also moderated by individual differences, such as age and facets of affinity for technology. More specifically, longer system response delays were compensated for by higher age and memory retention, which indicates the individual’s capacity to memorise and recall information. By contrast, longer system response delays resulted in lower recall performance with increasing self-reported competence for technology use. The latter might reflect the effects of increased frustration, as the user’s expectations for interacting with a fast-responding technology were not met. Although the study offers valuable hints about the underlying dynamics, the potential for broader conclusions is limited because it is based on a small student sample that is rather homogeneous with regards to age and gender. It is also worth examining whether the compensatory trend of increasing age persists in older age groups.

Building on the proven benefits of cognitive training for decelerating cognitive decline^8–10^, elderly participants represent a relevant target group for systematic memory training approaches. Computer-based settings provide accessibility for a broad audience, but often create difficulties for less technologically exposed generations. In this context, spoken dialogue systems become useful and offer a more natural way for humans to interact with these systems. However, to prevent the negative effects of spoken interactions, such as increased frustration due to slow or erroneous system reactions, it is crucial to create an adequate system performance, while bearing in mind the potential effects of system response times. Furthermore, as well-established research on cognitive aging shows, elderly people are a rather heterogeneous sample due to their rich history of individual experiences and variety of moderating influences, such as gender, education level, lifestyle and health status (e.g.^11–13^). This heightened interindividual variance becomes even more relevant when examining factors related to system development processes. With respect to memory performance, it is important to consider processes of decline associated with cognitive aging, such as a loss of cognitive speed^13,14^ and impaired cognitive control when facing external interference^15^. Due to the accompanying age-related changes, examining individual memory retention plays a key role in the interaction with technical systems as well.

Considering the evidence presented above, this study addresses the cognitive effects of delayed system response in intelligence voice-controlled technology in the elderly population. Within a dialogue-based memory training scenario with a virtual agent, influences of system response delay on memory performance are examined while considering user-related characteristics as moderators. In line with previous research, user frustration and perceived system quality are also assessed for the purposes of comparability.

From the outlined findings the hypothesis emerges that memory performance decreases in the older age group as system response delays increase (**H1**). Moreover, individual factors, such as memory retention, age, gender and facets of affinity for technology, are expected to affect this relationship (**H2**).

## Methods

### Participants

A sample of 62 native German-speaking elderly people (*M* = 69.03 years, *SD* = 5.48, range: 60–81, 57% female) participated in this study. The majority (91%) were retired from their jobs for at average 8.38 years (*SD* = 5.91, range: 1–22), and 71% held an academic degree. Moreover, 90% reported no previous experience with comparable training settings. A monetary reward of 5€ was granted as compensation, and ethical approval was obtained from the ethics committee of the Faculty of Humanities at the Technische Universität (TU) Chemnitz. In addition, institutional approval was obtained from the Managing Director of the Institute for Media Research at the TU Chemnitz. All participants provided informed consent and the research was performed in accordance with the relevant guidelines and regulations outlined in Standard 8 of the Ethical Principles and Code of Conduct for Psychologists^16^.

### Design and scoring

The participants were asked to memorize three lists of German nouns with increasing list lengths (five, seven and nine words) after receiving a dialogue-based memory training with a virtual agent. Table 1 presents an overview of the variables inspected in the experimental setting.

**Table 1:** Overview of variables and related methods of measurement.

Variable	Classification	Measurement
System response delay	IV	Length of system response time in seconds
Recall performance	DV	Total number of correctly recalled words in test phase after training
Frustration	DV	Subscale “Frustration” of NASA-TLX^17^
System quality	DV	Subscale “Overall evaluation” of meCUE^18^
Training performance	MeV	Total number of correctly recalled words during training phase
Memory retention	MoV	Total number of correctly recalled words during baseline phase prior to training
Facets of affinity for technology	MoV	Subscales “Enthusiasm”, “Competence”, “Positive perception”, and “Negative perception” of TA-EG^19^
Gender	MoV	Question in demographics questionnaire
Age	MoV	Question in demographics questionnaire

*Note*. IV = Independent variable, DV = Dependent variable, MeV = Mediator variable, MoV = Moderator variable. For moderator variables, both direct and interaction effects were inspected.

In a multivariate regression design, the system response delays during the training session were continuously varied in equally distributed intervals between 0.5–5.5 s in steps of 0.5 s. Participants were assigned their delay length randomly, which served as an independent variable. The random assignment procedure ensured an almost equal distribution across levels of system response delay, but it did not balance other subject characteristics. The system response delay was defined as the system response time starting as soon as the participant stopped talking to the system and ended when the system provided a verbal response. This verbal response was generated by the system in reaction to the participant’s prior dialog.

Recall performance was the main dependent variable that consisted of the accumulated number of correctly recalled words during the three word lists in the test pase without considering the correct word order. As an additional dependent variable, the participants’ frustration levels were measured using the continuous NASA-TLX subscale *frustration*^17^, which ranged from ‘very low’ to ‘very high’ with 20 gradual levels in between. The meCUE^18^ provided a further dependent variable through the continuous evaluation of overall perceived *system quality*. This variable was measured using 20 gradual levels that ranged from ‘bad’ (−5) to ‘good’ (5).

Controlling for user-related influences on memory performance, three word lists with five, seven and nine words before the memory training were considered the baseline for *memory retention*. Since this characteristic was assumed to affect the relationship between system response delay and recall performance, it was considered a moderating variable. According to^19^ and^20^, a moderator variable is a third variable that clarifies the conditions under which two variables are related to each other and is included in the analysis as an interaction effect. In contrast, training performance was considered as mediating variable, which is a third variable that affects the generative mechanism (i.e., how or why two variables are related)^19,20^. Since the system response delay was manipulated during the training, it was assumed that it would affect training performance, which would also affect recall performance. During the outlined baseline, training and test phases, different word lists were used to avoid carry-over effects.

The participants’ affinity for technology, which is assumed to reflect a positive attitude, enthusiasm and trust for technology, was obtained using the standardized questionnaire TA-EG^21^. According to the TA-EG authors, affinity for technology is accompanied by interest in and acceptance of technology and exerts a positive influence on knowledge of and experience with technology. The overall construct is comprised of four analytically validated dimensions that are assessed by the following subscales. *Enthusiasm for using technology* (α = 0.842) (hereafter enthusiasm) refers to information about and ownership of new technical devices, while *subjective competence in using technology* (α = 0.789) (hereafter competence) involves knowledge and use of technical devices. *Perception of positive effects of technology* (α = 0.722) (hereafter positive perception) and *perception of negative effects of technology* (α = 0.747) (hereafter negative perception) refer to consequences of using technical devices, such as increased independence (+) or stress (−). Each of the corresponding items was assessed on a five-point Likert scale from ‘fully applies’ to ‘does not apply at all’. The sum scores of the outlined subscales were also included as moderator variables. Additional moderating effects were expected to arise from age and gender.

### Materials

#### Word lists

Before conducting the study, a separate online-study with 99 two-syllable German nouns, derived from the word pool used by^22^, was conducted to determine 63 suitable words for the baseline (21), training (21) and test (21) phase. A total of 82 volunteers (*M* = 35.98 years, *SD* = 12.02, 71% female) participated in this pre-study, most of which were in the workforce (63%) or studying (26%). The majority held an academic degree (72%), and only 8.5% were non-native German speakers, but all participants had been speaking the language for at least 18 years. Not all participants completed the entire questionnaire, but for each chosen word, between 53 and 62 participants (*M* = 57.23, *SD* = 1.71) provided scale ratings.

The words were presented in a randomized order during the questionnaire and had to be rated on nine-point Likert-scales for difficulty (1 = ‘easy’ and 9 = ‘difficult’), concreteness (1 = ‘abstract’ and 9 = ‘concrete’) and emotionality (1 = ‘positive’ and 9 = ‘negative’). The most neutral words were defined in line with the scale means (*M* = 5, *SD* = 2), resulting in values between three and seven. Since only 34 words fell within this range, another 29 words were selected with a mean below or above the defined span in one or two of the subscales. Words with a mean outside of the range of 5 ± 2 in two subscales were only used during the training phase. Moreover, the majority of words in the baseline phase, and all of the words in the test phase were within this range. For instance, in the case of emotionality, only four words with a positive valence were outside the range: ‘donation’, ‘trip’, ‘applause’ and ‘gift’. Since all of the participants received the same words at the same time during the session, a constant influence of potential valence was assumed. Table 2 presents the resulting mean scale ratings and standard deviations (SDs) for the difficulty, concreteness and emotionality of the word lists used during the baseline, training and test phase. The word frequency ratings showed an average value of 11.30 (*SD* = 1.26), indicating medium use in the German language.

**Table 2:** Descriptive statistics for word list difficulty, concreteness, and emotionality in baseline, training, and test phase.

	Difficulty		Concreteness		Emotionality	
	*M*	*SD*	*M*	*SD*	*M*	*SD*
Baseline	3.54	0.66	6.05	1.07	4.12	0.65
Training	2.95	0.37	6.99	0.65	3.86	0.95
Test	3.81	0.54	5.60	0.80	4.00	0.77

*Note*. *M* = mean, *SD* = standard deviation; Descriptive indices were computed across all 21 words in each the baseline, training, and test phase. Each phase consists of three lists of words with different list length, one for each five, seven, and nine words.

#### Training characteristics

The training setting was based on the well-established method of loci^23^, which is also known as the ‘memory palace technique’. This method is comprised of a mnemonic device that links the content to be memorized with a spatial location that exists in a defined area. In this scenario, the participants were confronted with a virtual agent in a living room who asks them to memorise words by putting them on furniture or other objects in the space. In line with the training goal of developing transferrable skills for everyday use, this procedure involved fading instructional support according to the guidance-fading principle^24^.

**Figure 1:**
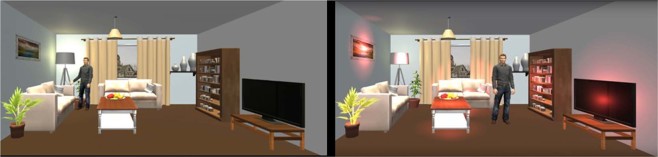
Virtual agent in the living room scenario. *Left*: Scene without highlights and agent walking around during presentation of first word list. *Right*: Scene with red highlights and static agent during second word list.

As depicted in Fig. 1, the presentation of the first word list in the training phase featured a fully furnished living room with the virtual agent walking around, pointing at five relevant objects (i.e., plant, lamp, table, book shelf, TV) and creating links between the words and objects. The objects were highlighted by a red light one after another when the word should be recalled. The second word list was also presented in the living room, but the seven relevant objects were highlighted simultaneously from the beginning and the participants were required to create links between the words and objects by themselves. For the presentation of the third word list, all of the furniture was removed, and the virtual agent was placed in an empty outline of a room. The participants were asked to imagine their own living room, name nine objects within, and link those objects to the words presented in the next step. After each complete word list recall, they received feedback on the number of words they recalled correctly.

#### Technical setup

**Figure 2:**
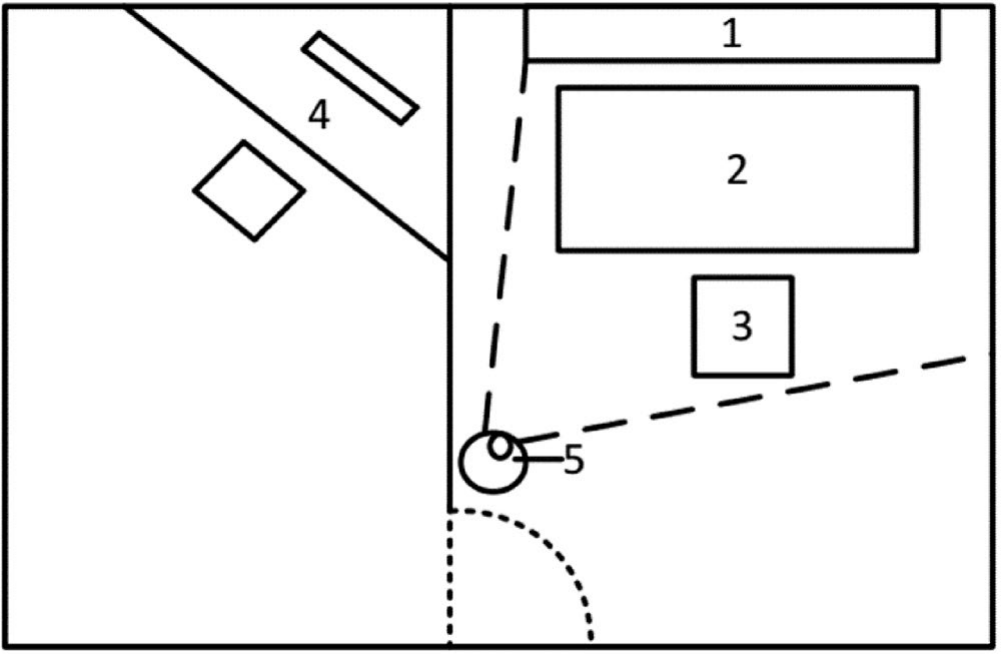
Technical setup. 1 = presentation screen, 2 = participant working table, 3 = participant seating area, 4 = experimenter working table, 5 = camera for audio recording (extension of^7^).

The training scenario required two adjacent rooms with workspaces for the experimenter and participant. The room was arranged based on the visual outline presented in Fig. 2. A Samsung Full HD TV with a display resolution of 1080p served as the presentation screen, and it was connected to a standard Windows 7 desktop computer for the experimenter with an HDMI cable. Auditory participant-related signals originated from the microphone in a Logitech QuickCam Pro 5000 and were conveyed to the experimenter via headphones. The camera had a resolution of 480p, but the images were not saved and were only used for additional visual control.

This study applies the Wizard-of-Oz technique^25^, which involves simulating system interactions via experimenter-controlled actions and is a common method for testing spoken dialogue systems in the early stages of development. Based on existing design criteria for Wizard-of-Oz studies^26–28^, the experimenter was required to select dialogue options from a set of predefined response paths in reaction to the participants’ spoken responses. A software framework was designed specifically for this purpose to ensure that the intended delay length was included in the system response. All potential verbal and behavioural responses of the virtual agent were represented in a set of videos that could be linked to each other with an arbitrarily long delay in between. The virtual agent itself was designed with the Virtual Human Toolkit^29^ and animated in the living room scenes with Unity. When a video was presented to the participant, the experimenter could choose the next video from a predefined selection based on the participant’s response by clicking the related visual button on the screen.

**Figure 3:**
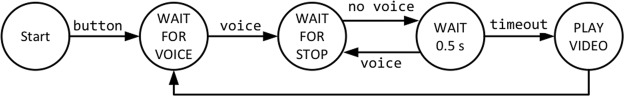
Graphical outline of system behaviour (extension of^7^). The figure shows the example for a minimum system response delay of 0.5 s.

An amplitude-based voice activation detector (VAD) was used to monitor the participants’ verbal responses and provide the basis for timing. As outlined in Fig. 3, for each participant the system started in a defined start state and could only precede if the experimenter activated a button in the on-screen menu. During this initial change of state, the length of system response delay was chosen randomly and subsequently used for all system interactions with that participant. In the state WAIT FOR VOICE, the system waited for the output of the VAD and switched to the state WAIT FOR STOP, when the participant’s voice was detected. After the participant stopped talking, the system moved into a waiting state and the intended delay was triggered. The experimenter had at minimum 0.5 s to select an adequate system response from a displayed set of logical possibilities. If no selection occurred within the available time span, the previous utterance of the Wizard-of-Oz system was repeated to ensure correct system response times. If the participant paused during speaking, the system could change back to the WAIT FOR STOP state to avoid unintentionally starting the system response delay. After successfully choosing a system response, the remaining delay had to pass before the next video could be played, and the system moved back to the WAIT FOR VOICE state.

### Procedure

After the welcoming statements, each session began by obtaining the participants’ formal consent. Then, the participants provided basic demographic data and completed the TA-EG questionnaire^21^ online (approximately 10 min). To assess the individual baseline of memory retention, the participants were required to memorise and recall three lists of words. The first list consisted of five words, the second list consisted of seven words, and the third list consisted of nine words. The lists were presented one after another by a computer-generated voice. The verbal presentation was accompanied by a white screen and followed by neutral feedback on the number of correctly recalled words (approximately 5 min). In the subsequent training phase, the participants were introduced to the previously described method of loci with decreasing instructional guidance over three word lists with one list of each five, seven, and nine words that immediately followed each other (approximately 25 min in total). Although the session structure was fixed for all participants, there was a certain amount of interindividual variation in terms of how long they saw the depicted scenes and the length of the response time. Both depended on the assigned system response delay and the comprehensibility of the participants’ answers. For example, some participants could recall more words than others and others needed more time to name the objects in their own living rooms during the presentation of the third word list in the training phase. After completing the training phase, another three word lists with one list for each five, seven and nine words were introduced to determine the participants’ recall performance and give them an opportunity to apply the method of loci (approximately 5 min). These words were presented verbally in the same manner as the word lists in the baseline phase with similar neutral performance-related feedback. Finally, the participants completed the NASA-TLX^17^ and meCUE^18^ online questionnaires (approximately 10 min), received their monetary compensation for participating in the experiment and were debriefed.

### Analysis

**Figure 4:**
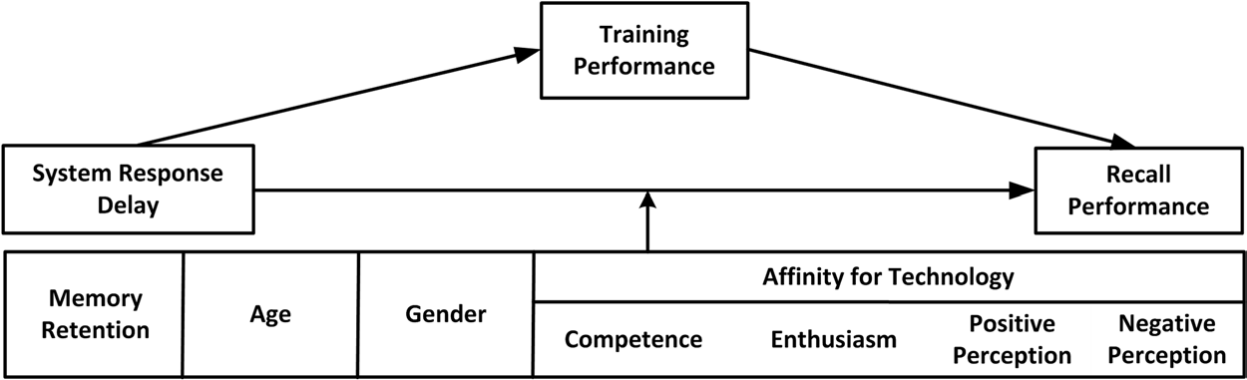
Path model with direct (main) effects, mediation effect, and moderation (interaction) effects for recall performance. Corresponding models were used for frustration and perceived system quality (extension of^7^). Affinity for technology was included with the separate subscales of enthusiasm, competence, positive perception, and negative perception.

A path model analysis was conducted to inspect effects on recall performance using the *lavaan* package^30^ in R (see Fig. 4). In addition to the direct influence of the system response delay on recall performance during the test phase, a mediation effect of training performance and the moderation effects of individual characteristics (i.e., memory retention, age, gender and facets of affinity for technology) were included. Comparable models were used to analyse effects on frustration and perceived system quality.

All analyses operated on z-standardized variables to obtain standardized beta coefficients, and applied maximum likelihood estimation, an alpha level of 0.05 and a two-tailed test procedure. The Satorra-Bentler scaled-test statistic and robust standard errors were used to account for the non-normal distribution of the inspected dependent variables. The results were based on *n* = 52 observations due to missing data and exclusions related to uncompensated hearing difficulties, instructional violations and technical malfunctions of the system. A post hoc inspection of differences in the potentially moderating variables across various system response delays revealed no statistically significant differences for memory retention (*H*(10) = 6.812, *p* = 0.743), age (*H*(10) = 16.969, *p* = 0.075), gender (*H*(10) = 13.543, *p* = 0.195), enthusiasm (*H*(10) = 9.005, *p* = 0.532), competence (*H*(10) = 11.769, *p* = 0.301), positive perception (*H*(10) = 12.974, *p* = 0.225) and negative perception (*H*(10) = 7.770, *p* = 0.651).

## Results

### Recall performance

The results for the direct effects indicate that the participants achieved better recall performance with better training performance (β = 0.360, *SE* = 0.066, *p* < 0.001). There was no significant relationship between the system response delay and training performance and the system response delay had no significant effect on recall performance. Therefore, no significant mediation effect can be assumed. Moreover, a gender effect emerged as women performed better in word recall (β = − 0.387, *SE* = 0.109, *p* < 0.001). Participants with a less negative perception also attained higher recall scores (β = −0.249, *SE* = 0.106, *p* = 0.018). While contrary to **H1** the system response delay had no significant direct effect, several considerable interaction effects observed in this study support **H2**.

**Figure 5:**
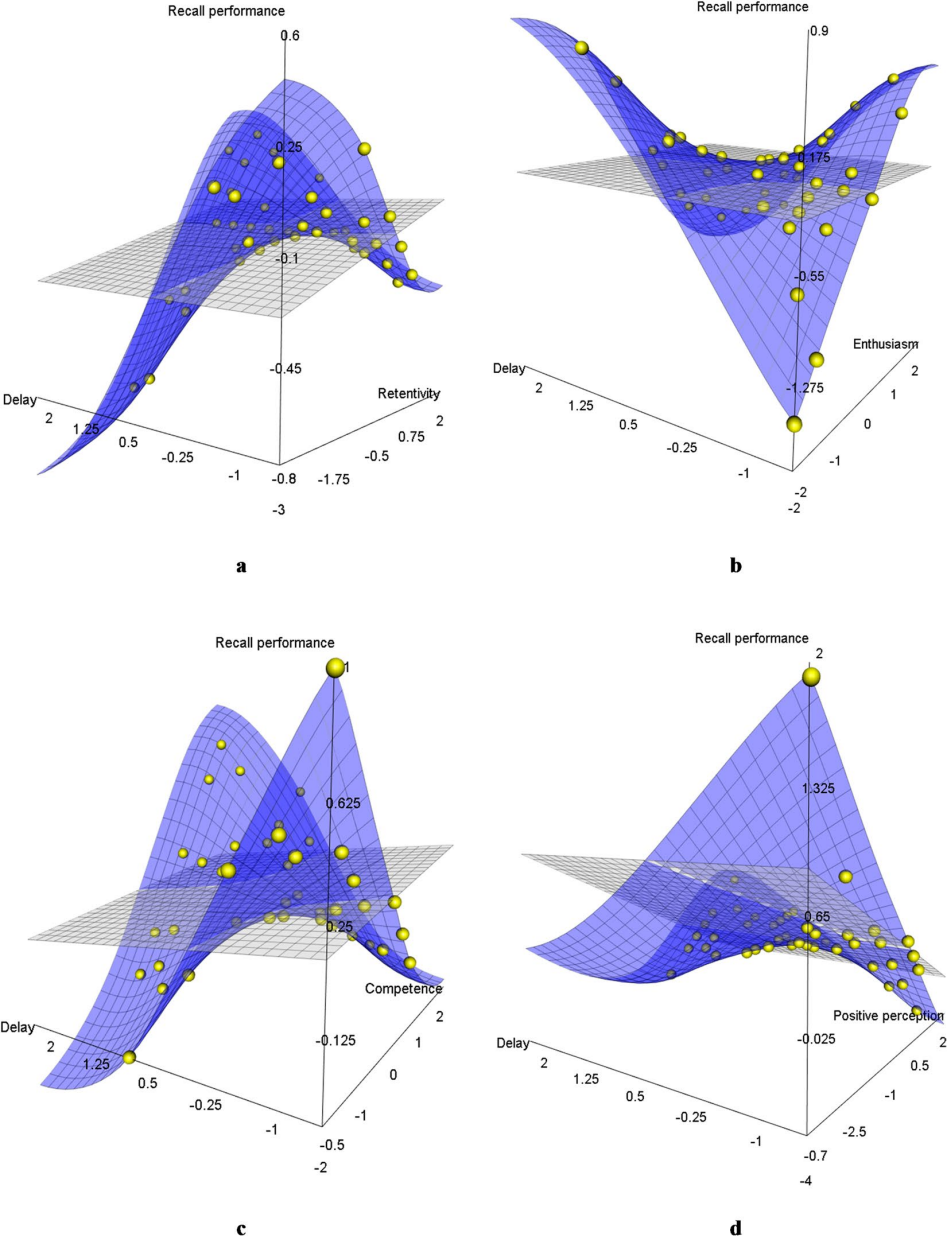
Moderation effects on the influence of system response delays on recall performance. (**a)** Memory retention, (**b)** Enthusiasm (subscale of affinity for technology), (**c**) Competence (subscale of affinity for technology), (**d**) Positive perception (subscale of affinity for technology). All axes represent z-values instead of original units for reasons of comparability. Each dot represents one participant (or more than one participant in case of overlapping data points).

Figure 5a suggests that higher memory retention could compensate for performance loss when system response delays increased (β = 0.279, *SE* = 0.109, *p* = 0.010). On a descriptive level, the displayed plane consists of an orthogonally linked U-shaped and inverted U-shaped progression, which reveal an inflection point at their medium level for both interacting factors. Similar compensation effects seemed to emerge with higher competence (see Fig. 5c) (β = 0.362, *SE* = 0.150, *p* = 0.016), and higher positive perception (see Fig. 5d) (β = 0.283, *SE* = 0.105, *p* = 0.007). In contrast, participants with higher enthusiasm seemed to achieve lower recall performance as the system response delays increased (β = −0.527, *SE* = 0.164, *p* = 0.001), which is indicated by the inverted progression of both orthogonal U-shaped curves presented in Fig. 5b.

**Figure 6:**
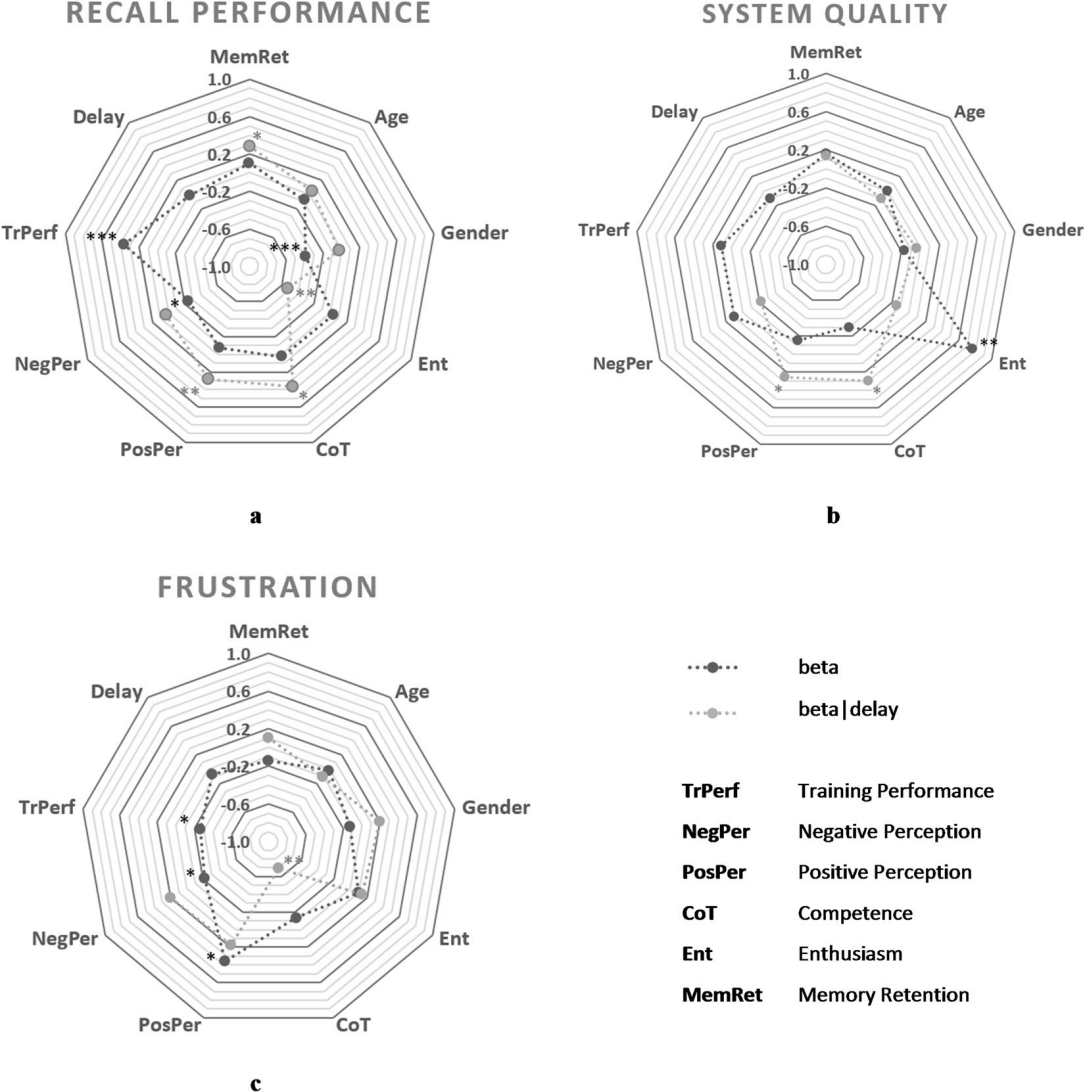
Summarized overview on the influence of direct (main) effects (dark grey) and moderation (interaction) effects (light grey). (**a**) Recall performance, (**b**) Perceived system quality, (**c**) Frustration. Dots represent standardized beta weights for direct effects (beta) and moderation effects (beta | delay). Asterisks indicate the level of significance (***p < 0.001, **p < 0.01, *p < 0.05).

Figure 6a summarizes the direct and interaction effects used in this model, which makes it possible to compare their strength and significance. In several cases, the individual characteristics of memory retention, competence, positive perception and enthusiasm seemed to exert stronger influence when considering the length of the system response delays compared to the strength of their separate influence. Overall, the model explained nearly 55% of the existing variance (R^2^ = 0.549).

### Perceived system quality

In terms of the direct effects on perceived system quality, participants with higher enthusiasm showed significantly better system ratings (β = 0.764, *SE* = 0.223, *p* = 0.001).

**Figure 7:**
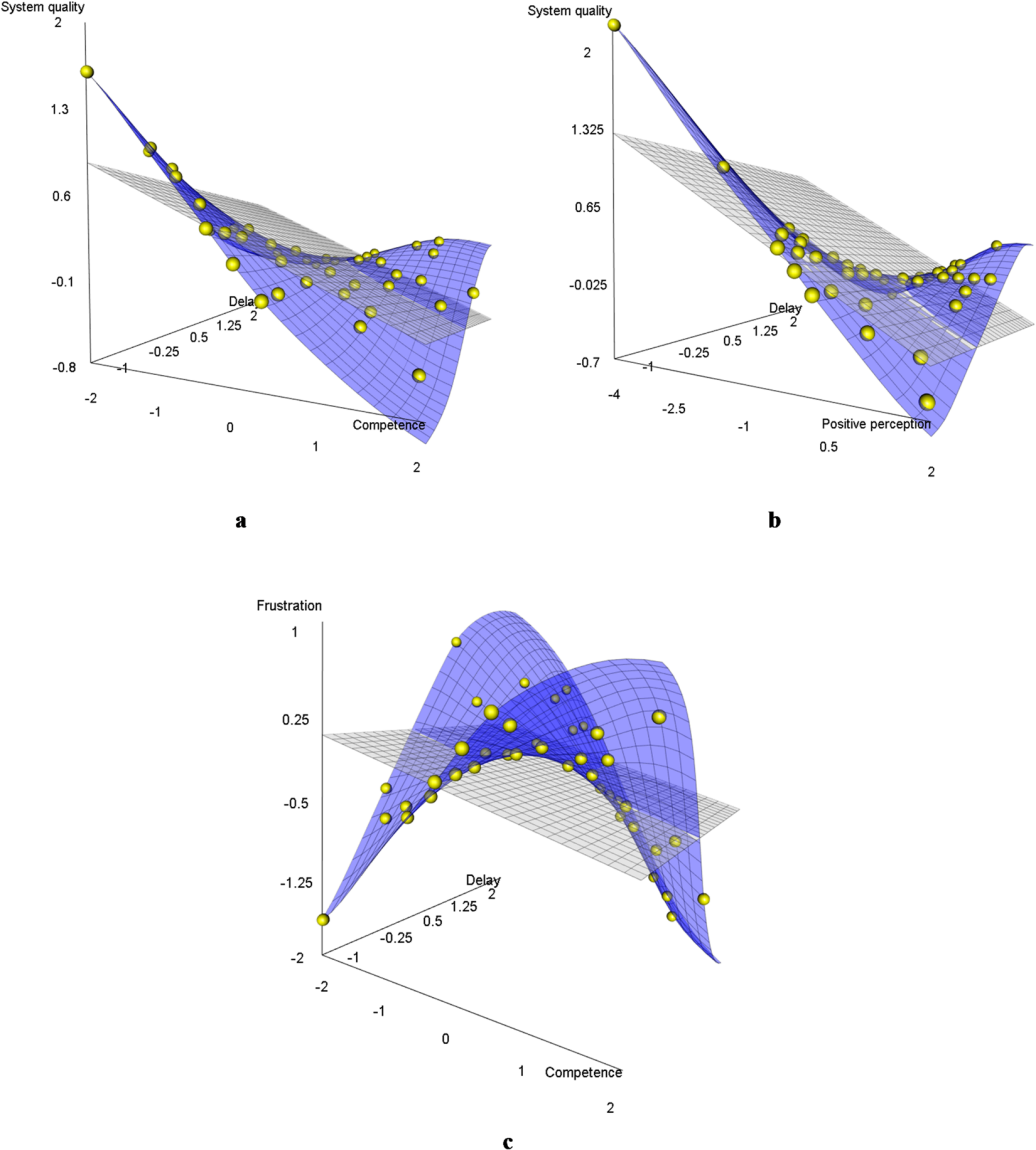
Moderation effects of competence for technology use and positive perception of technology (both subscales of affinity for technology) on the influence of delay on subjective ratings. (**a**,**b**) Perceived system quality, (**c**) Frustration. All axes represent z-values instead of original units for reasons of comparability. Each dot represents one participant (or more than one participant in case of overlapping data points).

Regarding the interaction effects, Fig. 7a,b show that higher competence (β = 0.299, SE = 0.142, *p* = 0.035) and higher positive perception (β = 0.259, *SE* = 0.120, *p* = 0.030) can compensate slightly for increased system response delays, although higher perceived system quality seems to be related to shorter system response delays. Compared to the recall performance model described above, the orthogonal U-shaped progressions are less prominent.

Figure 6b summarizes this pattern, which usually indicates equal strength between the direct and moderating effects in the sample, although occasionally in opposite directions. On one hand, the effects of competence and positive perception seem to gain significance when considering the system response delay. On the other hand, the reversed moderation effect of enthusiasm loses significance. Overall, the model explained 37% of the existing variance (R^2^ = 0.372).

### Frustration

Regarding the direct effects, the participants’ frustration levels increased when their positive perception was higher (β = 0.362, *SE* = 0.148, *p* = 0.015). Once again, increasing the system response delay seemed to have no direct effect on this dependent variable. In contrast, higher levels of negative perception (β = −0.216, *SE* = 0.107, *p* = 0.044) and higher training performance (β = −0.260, *SE* = 0.116, *p* = 0.025) resulted in lower levels of frustration.

In terms of the interaction effects (see Fig. 7c), higher competence resulted in lower levels of frustration as the system response delays increased (β = −0.697, *SE* = 0.208, *p* = 0.001). Similar to the descriptive pattern of recall performance described above, both interacting factors show an inflection point between the orthogonally linked U-shaped and the inverted U-shaped curve at their medium level.

The summary provided in Fig. 6c shows that the direct and moderating effects are more balanced. However, while the moderation effects of positive and negative perception seem to lack significance, the visual outline suggests that competence only exerts significant influence on reported frustration when the length of system response delays is considered. Overall, the model explained approximately 40% of the existing variance (R^2^ = 0.408).

## Discussion

**Figure 8:**
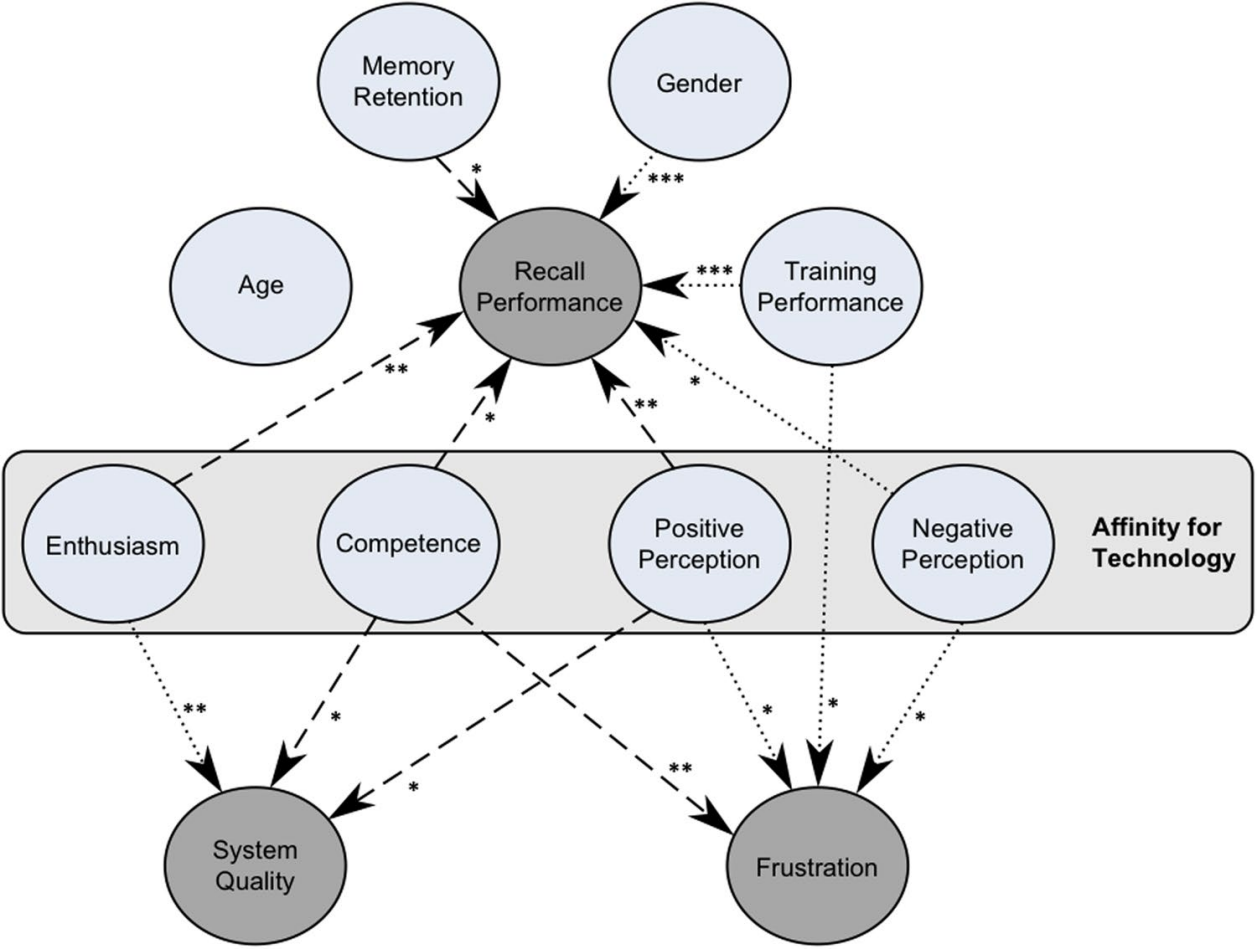
Summary of the pattern of effects across the path models for recall performance, system quality and frustration. Grey circles indicate dependent variables, blue circles indicate moderator and mediator variables. Dotted lines represent direct effects, dashed lines represent interaction effects with system response delay. Asterisks indicate the level of significance (***p < 0.001, **p < 0.01, *p < 0.05).

This study investigated the effects of system response delay on recall performance, frustration and perceived system quality in an elderly sample using a virtual memory training scenario, while simultaneously considering individual characteristics. Figure 8 summarizes the obtained pattern of direct effects (dotted lines) and interaction effects with system response delay (dashed lines). As displayed, higher training performance seemed to benefit recall performance and decrease frustration. The system response delay seemed to have no direct effect on recall performance, which contradicts the first hypothesis. In contrast, memory retention, gender and facets of affinity for technology showed several direct and interaction effects on recall performance, which supports the second hypothesis. In addition, these individual characteristics seemed to influence frustration and perceived system quality.

### Effects on cognitive performance

Comparing the outlined results to the effect pattern obtained by^7^ raises the question why the system response delay had no direct effect on recall performance. A first potential explanation relates to the heterogeneous sample of elderly participants that was used in this study. This assumption received further support by the moderating influences of individual characteristics, such as memory retention and facets of affinity for technology. Even participants who reported a high level of affinity for technology on either of the subscales were not comparable to people of younger ages simply because elderly populations have had less exposure to technology. The less frequent use of technology could mean that expectations for system perfection were less distinct, which could further contribute to the arising pattern of individual differences.

In addition to the compensating effects of higher *memory retention*, a second potential explanation hints on the benefits of a longer system response delay related to *segmentation*. Following the segmenting effect described in the multimedia learning literature, people should experience better performance when relevant information is segmented, which means that it is presented sequentially in meaningful and coherent units^31^. The existing research shows that inserting meaningful pauses into learning material releases working memory resources and provides additional time for processing relevant learning content (e.g.^32^). This segmented method of presenting information could compensate for the previously described reductions in cognitive abilities as people age.

### Facets of affinity for technology as moderating factors

Approaching the reported facets of affinity for technology in more detail, with increased system response delays a higher reported level of *enthusiasm* seemed to be harmful for cognitive performance. This could be explained by disappointment, as the participants expectations of immediate system reactions were not met. The resulting frustration could have impaired their performance^33^. In contrast, participants who reported less enthusiasm for technology might have had lower expectations for system functionality. Therefore, they were more focused on the beneficial effects of pauses that resulted from increased system response delays.

The participants who reported a more *positive perception* of technology also benefitted from higher system response delays in terms of recall performance. In this case, the resulting segmentation could have triggered positive emotions that improved performance^33^. This finding is supported by the reversed pattern in the of absence of positive perception. Furthermore, increased system response delays resulted in higher perceived system quality when the participants had a more positive perception of technology.

*Competence* is a manifold parameter that influences recall performance, frustration and perceived system quality. While a longer system response delay results in better recall performance and less frustration with increasing competence, it also elicits a decrease in perceived system quality. This pattern could be explainable by the heterogeneity of the participants, while raising the necessity to consider specific requirements in system development. Therefore, determining the optimal length of system response delays is a core challenge, especially when considering recall performance, frustration and perceived system evaluation simultaneously.

### Gender affects memory recall

The higher recall performance of elderly women aligns with the overall patterns in cognitive decline^12^ where women show better performance in tasks involving verbal components. In addition, traditional gender roles in the current elderly population might also be relevant in this setting. Following existing stereotypes, woman may characterize themselves as more verbally adept than men and perform accordingly. The fact that age does not have a direct effect on recall is supported by existing research showing that a variety of individual factors gain importance after the age of 60 (e.g.^11,13^).

### Implications, limitations and future research

In summary, the results of this study show the high impact of individual differences in older age and emphasizes the trade-offs between perceived system quality and support of recall performance based on higher affinity for technology. However, the study by^7^ suggests that this pattern might shift over time as the affinity for technology increases. A more systematic investigation might compare different age groups, such as adolescents, middle-aged adults and elderly people. With a sufficiently broad and balanced sample size across different age groups, valid conclusions on the underlying effects of age would be possible and would help to determine the optimal trade-offs in system response delays. Moreover, ensuring more balanced subsamples across system response delays would further help to strengthen the results obtained in this study.

Follow-up studies should include a standardized cognitive test for elderly participants to provide more comprehensive insights into the dynamics of cognitive decline. Furthermore, evidence shows that participants’ memory performance across different age groups is also affected by the valence and arousal of memorized content. For instance^34^, reported that older adults outperformed younger adults when memorizing positive stimuli and vice versa. On this account, using a more balanced word list with reference to valence and arousal would strengthen the conclusions beyond the effects of age. Finally, extending the amount of dialogue opportunities included in the training procedure and keeping track of participants’ frustration would help to overcome the existing limitations.

## Conclusion

Due to the high prevalence of interactive technology in modern societies, delayed responses in technical systems are omnipresent. This study presented a novel inspection of resulting influences beyond frustration and perceived system quality on the level of cognitive performance. Contrary to the common goal of increasing the speed of system reactions, the results suggest that longer system response times can have performance benefits for elderly user groups. The ambiguous effect patterns of cognitive benefits and subjective preferences related to system response times raises an ongoing challenge when designing computer-based training systems. The optimal trade-off between both dimensions needs to be explored in more detail in future research.

## Supplementary information

R-Script for creating and saving rotatable 3D-graphs and performing path model analyses

Supplementary Dataset 1

## Data Availability

The dataset that was used to perform the path model analyses and create rotatable versions of the moderation graphs (see Figs 5 and 7) is included in the Supplementary Information. Additional data can be requested from the corresponding author.
