# Supplementary material for: Effects of system response delays on elderly humans’ cognitive performance in a virtual training scenario
Source: Sci Rep. 2019 Jun 5;9:8291. doi: 10.1038/s41598-019-44718-x (PMC6549173; doi:10.1038/s41598-019-44718-x)
Supplement: Supplementary file 1 — R-Script for creating and saving rotatable 3D-graphs and performing path model analyses [file 41598_2019_44718_MOESM1_ESM.pdf]

Effects of system response delays on elderly humans' cognitive performance in a virtual training scenario

Maria Wirzberger[1,5], René Schmidt[2], Maria Georgi[3,4], Wolfram Hardt[2], Guido Brunnett[4], and Günter Daniel Rey[5]

[1]Max Planck Research Group "Rationality Enhancement", Max Planck Institute for Intelligent Systems, Max-Planck-Ring 4, 72076 Tübingen, Germany

[2]Computer Engineering, Faculty of Computer Science, TU Chemnitz, Straße der Nationen 62, 09111, Germany

[3]Information Science, Faculty of Languages, Literature and Culture, University of Regensburg, Universitätsstraße 31, 93053 Regensburg, Germany

[4]Computer Graphics and Visualization, Faculty of Computer Science, TU Chemnitz, Straße der Nationen 62, 09111 Chemnitz, Germany

[5]Psychology of Learning with Digital Media, Faculty of Humanities, TU Chemnitz, Straße der Nationen 12, 09111 Chemnitz, Germany

Maria Wirzberger and René Schmidt contributed equally

Corresponding author: Maria Wirzberger (maria.wirzberger@tuebingen.mpg.de)

```r
#################################################################################################
### R-Script for creating and saving rotatable 3D-graphs and performing path model analyses ###
###                                                                                         ###
### Dr. Maria Wirzberger                                                                    ###
### Max Planck Institute for Intelligent Systems Tübingen, Germany                          ###
### maria.wirzberger@tuebingen.mpg.de                                                       ###
###                                                                                         ###
### 07/02/2019                                                                              ###
#################################################################################################


# Load relevant libraries
library(lavaan)
library(car)
library(rgl)


# Load dataset
df_fin <- read.csv("G:/dataset.csv") # replace by adequate paths to dataset file

### Description of included variables
# "VP_Nummer" --> participant number
# "Delay_Dauer" --> length of delay in s
# "Alter" --> age in years
# "Geschlecht" --> gender (1 = male, 2 = female)
# "Geistige_Anforderung" --> mental demands score from NASA-TLX
# "Koerperliche_Anforderung" --> physical demands score from NASA-TLX
# "Zeitliche_Anforderung" --> temporal demands score from NASA-TLX
# "Ausfuehrung_Aufgaben" --> task execution score from NASA-TLX
# "Anstrengung" --> effort score from NASA-TLX
# "Frustration" --> frustration score from NASA-TLX
# "TA_Begeisterung_ges" --> enthusiasm score from affinity for technology questionnaire (TA-EG)
# "TA_Kompetenz_ges" --> competence score from affinity for technology questionnaire (TA-EG)
# "TA_pos_Technikfolgen_ges" --> positive perception of technology score from affinity for technology questionnaire (TA-EG)
# "TA_neg_Technikfolgen_ges" --> negative perception of technology score from affinity for technology questionnaire (TA-EG)
# "IP_Produktwahrnehmung_ges" --> product perception score from meCUE
# "IP_Nutzeremotion_ges" --> user emotions score from meCUE
# "IP_Konsequenzen_ges" --> use consequences score from meCUE
# "IP_Gesamturteil" --> system quality score from meCUE
# "T_ges" --> memory performance in test runs (recall performance; accumulated proportionate amount of correctly recalled words without order)
# "B_ges" --> memory performance in baseline runs (retentivity; accumulated proportionate amount of correctly recalled words without order)
# "U_ges" --> memory performance in training runs (training performance; accumulated proportionate amount of correctly recalled words without order)
# "Delay_Dauer_z" --> length of delay in s, z-standardized
# "Alter_z" --> age in years, z-standardized
# "Geschlecht_z" --> gender, z-standardized
# "TA_Begeisterung_ges_z" --> enthusiasm score from affinity for technology questionnaire (TA-EG), z-standardized
# "TA_Kompetenz_ges_z" --> competence score from affinity for technology questionnaire (TA-EG), z-standardized
# "TA_neg_Technikfolgen_ges_z" --> negative perception of technology score from affinity for technology questionnaire (TA-EG), z-standardized
# "TA_pos_Technikfolgen_ges_z" --> positive perception of technology score from affinity for technology questionnaire (TA-EG), z-standardized
# "T_ges_z" --> memory performance in test runs (recall performance), z-standardized
# "B_ges_z" --> memory performance in baseline runs (retentivity), z-standardized
# "U_ges_z" --> memory performance in training runs (training performance), z-standardized
# "IP_Gesamturteil_z" --> system quality score from meCUE, z-standardized
# "IP_Konsequenzen_ges_z" --> use consequences score from meCUE, z-standardized
# "IP_Nutzeremotion_ges_z" --> user emotions score from meCUE, z-standardized
# "IP_Produktwahrnehmung_ges_z" --> product perception score from meCUE, z-standardized
# "Geistige_Anforderung_z" --> mental demands score from NASA-TLX, z-standardized
# "Koerperliche_Anforderung_z" --> physical demands score from NASA-TLX, z-standardized
```

```r
# "Zeitliche_Anforderung_z" --> temporal demands score from NASA-TLX, z-standardized
# "Ausfuehrung_Aufgaben_z" --> task execution score from NASA-TLX, z-standardized
# "Anstrengung_z" --> effort score from NASA-TLX, z-standardized
# "Frustration_z" --> frustration score from NASA-TLX, z-standardized
# "product_base_delay_z" -->  product term for moderation effect retentivity*delay, z-standardized
# "product_age_delay_z" -->  product term for moderation effect age*delay, z-standardized
# "product_gender_delay_z" --> product term for moderation effect gender*delay, z-standardized
# "product_begeisterung_delay_z" --> product term for moderation effect enthusiasm*delay, z-standardized
# "product_kompetenz_delay_z" --> product term for moderation effect competence*delay, z-standardized
# "product_positiv_delay_z" --> product term for moderation effect positive perception of technology*delay, z-standardized
# "product_negativ_delay_z" --> product term for moderation effect negative perception of technology*delay, z-standardized
# "plane_Delay_Base_z" --> moderation plane of retentivity*delay for recall performance, z-standardized
# "plane_Delay_Beg_z" --> moderation plane of enthusiasm*delay for recall performance, z-standardized
# "plane_Delay_Komp_z" --> moderation plane of competence*delay for recall performance, z-standardized
# "plane_Delay_Pos_z" --> moderation plane of positive perception of technology*delay for recall performance, z-standardized
# "plane_Delay_Komp_IP_z" --> moderation plane of competence*delay for system quality, z-standardized
# "plane_Delay_Pos_IP_z" --> moderation plane of positive perception of technology*delay for system quality, z-standardized
# "plane_Delay_Komp_Frust_z" --> moderation plane of competence*delay for frustration, z-standardized


# Build and save rotatable graphs (adjust path where graphs are saved if necessary)
# Change to 'fit="'"linear" and 'residuals=TRUE' to get linear plane with projections of residuals to surface
scatter3d(plane_Delay_Base_z ~ Delay_Dauer_z + B_ges_z, data=df_fin, xlab="Delay", ylab="Recall performance", zlab="Retentivity", fit=c("smooth", "linear"), axis.scales=TRUE, axis.ticks=TRUE, residuals=FALSE, fogtype="none", surface=TRUE, fill=TRUE, surface.col=c("blue","grey"), surface.alpha=0.4, axis.col=c("black","black","black"))
rgl.snapshot("G:/Recall_Retentivity_Delay.png")

scatter3d(plane_Delay_Beg_z ~ Delay_Dauer_z + TA_Begeisterung_ges_z, data=df_fin, xlab="Delay", ylab="Recall performance", zlab="Enthusiasm", fit=c("smooth", "linear"), axis.scales=TRUE, axis.ticks=TRUE, residuals=FALSE, fogtype="none", surface=TRUE, fill=TRUE, surface.col=c("blue","grey"), surface.alpha=0.4, axis.col=c("black","black","black"))
rgl.snapshot("G:/Recall_Enthusiasm_Delay.png")

scatter3d(plane_Delay_Komp_z ~ Delay_Dauer_z + TA_Kompetenz_ges_z, data=df_fin, xlab="Delay", ylab="Recall performance", zlab="Competence", fit=c("smooth", "linear"), axis.scales=TRUE, axis.ticks=TRUE, residuals=FALSE, fogtype="none", surface=TRUE, fill=TRUE, surface.col=c("blue","grey"), surface.alpha=0.4, axis.col=c("black","black","black"))
rgl.snapshot("G:/Recall_Competence_Delay.png")

scatter3d(plane_Delay_Pos_z ~ Delay_Dauer_z + TA_pos_Technikfolgen_ges_z, data=df_fin, xlab="Delay", ylab="Recall performance", zlab="Positive perception", fit=c("smooth", "linear"), axis.scales=TRUE, axis.ticks=TRUE, residuals=FALSE, fogtype="none", surface=TRUE, fill=TRUE, surface.col=c("blue","grey"), surface.alpha=0.4, axis.col=c("black","black","black"))
rgl.snapshot("G:/Recall_Positive_Delay.png")

scatter3d(plane_Delay_Komp_IP_z ~ Delay_Dauer_z + TA_Kompetenz_ges_z, data=df_fin, xlab="Delay", ylab="System quality", zlab="Competence", fit=c("smooth", "linear"), axis.scales=TRUE, axis.ticks=TRUE, residuals=FALSE, fogtype="none", surface=TRUE, fill=TRUE, surface.col=c("blue","grey"), surface.alpha=0.4, axis.col=c("black","black","black"))
rgl.snapshot("G:/Quality_Competence_Delay.png")

scatter3d(plane_Delay_Pos_IP_z ~ Delay_Dauer_z + TA_pos_Technikfolgen_ges_z, data=df_fin, xlab="Delay", ylab="System quality", zlab="Positive perception", fit=c("smooth", "linear"), axis.scales=TRUE, axis.ticks=TRUE, residuals=FALSE, fogtype="none", surface=TRUE, fill=TRUE, surface.col=c("blue","grey"), surface.alpha=0.4, axis.col=c("black","black","black"))
rgl.snapshot("G:/Quality_Positive_Delay.png")

scatter3d(plane_Delay_Komp_Frust_z ~ Delay_Dauer_z + TA_Kompetenz_ges_z, data=df_fin, xlab="Delay", ylab="Frustration", zlab="Competence", fit=c("smooth", "linear"), axis.scales=TRUE, axis.ticks=TRUE, residuals=FALSE, fogtype="none", surface=TRUE, fill=TRUE, surface.col=c("blue","grey"), surface.alpha=0.4, axis.col=c("black","black","black"))
rgl.snapshot("G:/Frustration_Competence_Delay.png")
```

```
# Analyze data with path models
# Recall performance
model_1A_z <- 'T_ges_z ~ a*Delay_Dauer_z + b*B_ges_z + c*Alter_z + d*Geschlecht_z + e*TA_Begeisterung_ges_z + 
f*TA_Kompetenz_ges_z + g*TA_pos_Technikfolgen_ges_z + h*TA_neg_Technikfolgen_ges_z + i*U_ges_z + 
j*product_base_delay_z + k*product_age_delay_z + l*product_gender_delay_z + m*product_begeisterung_delay_z + 
n*product_kompetenz_delay_z + o*product_positiv_delay_z + p*product_negativ_delay_z
            U_ges_z ~ z*Delay_Dauer_z
            al := a*z
            total := a + b + c + d + e + f + g + h + i + j + k + l + m + n + o + p + (a*z)'
fit_1A_z <- sem(model_1A_z, data = df_fin, estimator="mlm", se="robust", test="satorra.bentler", mimic="Mplus")
summary(fit_1A_z, fit.measures=FALSE, standardized=FALSE, rsquare=TRUE)

# System quality
model_2A <- 'IP_Gesamturteil_z ~ a*Delay_Dauer_z + b*B_ges_z + c*Alter_z + d*Geschlecht_z + 
e*TA_Begeisterung_ges_z + f*TA_Kompetenz_ges_z + g*TA_pos_Technikfolgen_ges_z + h*TA_neg_Technikfolgen_ges_z 
+ i*U_ges_z + j*product_base_delay_z + k*product_age_delay_z + l*product_gender_delay_z + 
m*product_begeisterung_delay_z + n*product_kompetenz_delay_z + o*product_positiv_delay_z + 
p*product_negativ_delay_z
            U_ges_z ~ z*Delay_Dauer_z
            al := a*z
            total := a + b + c + d + e + f + g + h + i + j + k + l + m + n + o + p + (a*z)'
fit_2A <- sem(model_2A, data = df_fin, estimator="mlm", se="robust", test="satorra.bentler", mimic="Mplus")
summary(fit_2A, fit.measures=FALSE, standardized=FALSE, rsquare=TRUE)

#Frustration
model_3A <- 'Frustration_z ~ a*Delay_Dauer_z + b*B_ges_z + c*Alter_z + d*Geschlecht_z + 
e*TA_Begeisterung_ges_z + f*TA_Kompetenz_ges_z + g*TA_pos_Technikfolgen_ges_z + h*TA_neg_Technikfolgen_ges_z 
+ i*U_ges_z + j*product_base_delay_z + k*product_age_delay_z + l*product_gender_delay_z + 
m*product_begeisterung_delay_z + n*product_kompetenz_delay_z + o*product_positiv_delay_z + 
p*product_negativ_delay_z
            U_ges_z ~ z*Delay_Dauer_z
al := a*z
total := a + b + c + d + e + f + g + h + i + j + k + l + m + n + o + p + (a*z)'
fit_3A <- sem(model_3A, data = df_fin, estimator="mlm", se="robust", test="satorra.bentler", mimic="Mplus")
summary(fit_3A, fit.measures=FALSE, standardized=FALSE, rsquare=TRUE)
```
